# High Glucose with Insulin Induces Cell Cycle Progression and Activation of Oncogenic Signaling of Bladder Epithelial Cells Cotreated with Metformin and Pioglitazone

**DOI:** 10.1155/2019/2376512

**Published:** 2019-01-09

**Authors:** Daejin Kim, Byul-Nim Ahn, YeongSeok Kim, Dae Young Hur, Jae Wook Yang, Ga Bin Park, Jung Eun Jang, Eun Ju Lee, Min Jeong Kwon, Tae Nyun Kim, Mi Kyung Kim, Jeong Hyun Park, Byoung Doo Rhee, Soon Hee Lee

**Affiliations:** ^1^Department of Anatomy, Inje University College of Medicine, Busan 614-735, Republic of Korea; ^2^T2B Infrastructure Center for Ocular Disease, Inje University Busan Paik Hospital, Busan, Republic of Korea; ^3^Department of Ophthalmology, Inje University College of Medicine, Inje University Busan Paik Hospital, Busan, Republic of Korea; ^4^Department of Biochemistry, Kosin University College of Medicine, Busan 49267, Republic of Korea; ^5^Department of Internal Medicine, College of Medicine, Inje University, Busan, Republic of Korea

## Abstract

Metformin and pioglitazone are two commonly prescribed oral hypoglycemic agents for diabetes. Recent evidence suggests that these drugs may contribute to bladder cancer. This study investigated molecular mechanism underlying effects of metformin and pioglitazone in bladder epithelial carcinogenesis in type 2 diabetes. The cells derived from human bladder epithelial cells (HBlEpCs) were treated with metformin or pioglitazone with high glucose and insulin. Cell viability and proliferation were evaluated using the 3-(4,5-dimethylthiazol-2-yl)-2,5-diphenyltetrazolium bromide assay and a bromodeoxyuridine incorporation assay, respectively, while cell cycle regulatory factors and oncogene expression were analyzed using western blotting. Metformin or pioglitazone suppressed cell viability concentration and time dependently, which was reversed by exposure to high glucose with or without insulin. Prolonged exposure to high glucose and insulin enhanced cyclin D, cyclin-dependent kinase 4 (Cdk4), and Cdk2 expression and suppressed cyclin-dependent kinase inhibitors p21 and p15/16 in HBlEpC cotreated with pioglitazone and metformin. Levels of tumor suppressor proteins p53 and cav-1 were downregulated while those of the oncogenic protein as c-Myc were upregulated under high glucose and insulin supplementation in HBlEpC cotreated with pioglitazone and metformin. Prolonged exposure to high glucose with or without insulin downregulated B cell lymphoma 2-associated X (Bax) and failed to enhance the expression of extracellular signal-regulated kinase (ERK) and p38 mitogen-activated protein kinase (p38MAPK) in drug-treated cells. These results suggest that hyperglycemic and insulinemic conditions promote cell cycle progression and oncogenic signaling in drug-treated bladder epithelial cells and uncontrolled hyperglycemia and hyperinsulinemia are probably greater cancer risk factors than diabetes drugs.

## 1. Introduction

The association between diabetes and cancer may be explained in part by the shared risk factors associated with the two diseases such as aging, obesity, physical inactivity, and socioeconomic status and the metabolic abnormalities related to diabetes such as hyperglycemia and hyperinsulinemia [1]. Significant evidence exists linking diabetes with breast, colon, liver, and pancreatic cancers [2–5]. In contrast, the link between diabetes and bladder cancer is more controversial [6–10]. Metformin and pioglitazone are two commonly prescribed oral hypoglycemic agents for patients with diabetes. Recent evidence suggests that these drugs may affect the occurrence of a bladder cancer.

In the absence of contraindications, metformin alone or in combination with other drugs is considered the first-choice oral treatment of type 2 diabetes [11]. Metformin inhibits the proliferation of various types of cancer cells [12, 13] and enhances the efficiency of chemotherapy through tumor necrosis factor- (TNF-) related apoptosis-inducing ligand- (TRAIL-) induced apoptosis in human bladder cancer cells [14]. Metformin has been shown to suppress bladder cancer cell proliferation and potentiate cancer cell apoptosis via the mechanistic target of rapamycin (mTOR) pathway [14, 15]. In contrast to these studies, several meta-analyses did not show an association between metformin use and protection against bladder cancer risk [16–18]. These results suggest that metformin is less effective in preventing bladder cancer compared to other types of cancers.

Pioglitazone, a peroxisome proliferator-activated receptor-*γ* (PPAR*γ*) agonist with some activity against PPAR*α*, is a member of the thiazolidinedione class of antihyperglycemic drugs. Pioglitazone exerts its effects by increasing insulin sensitivity by the activation of PPAR*γ*. PPARs are nuclear hormone receptors that have a variety of regulatory functions in adipogenesis, cellular differentiation, and metabolic homeostasis [19]. PPAR*γ* is mainly expressed in white adipose tissue where it modulates lipid metabolism as well as insulin sensitivity. The synthetic PPAR*γ* agonist thiazolidinedione (TZD) potentiates PPAR*γ* function to improve glucose tolerance and restore the function of *β* cells [20–22]. Treatment of tumor cells with PPAR*γ* agonists was found to induce cell cycle arrest or stimulate apoptosis via the induction of p21 or downregulation of cyclin D1 [23–25]. PPAR*γ* activation in the presence of the retinoblastoma protein (RB) causes cell cycle arrest at the G1 phase, whereas in the absence of RB, cells accumulate at G2/M, leading to apoptosis [26]. In contrast to the anticancer effects of PPAR*γ* agonists, PPAR*γ* stimulation leads to the development of colon cancer in mouse models [27, 28]. In addition, pioglitazone use has been linked to increased risk of bladder cancer at high cumulative doses and following exposure for more than 2 years [29–31]. Therefore, the French and German Agencies for the Safety of Health Products suspended the use of pioglitazone in June 2011 because the overall risks associated with the drug outweigh its benefits [32]. The US Food and Drug Administration (FDA) did not suspend the market authorization but issued a black box warning for bladder cancer risk [33]. The Scientific Advisory Group in Diabetes/Endocrinology of European Medicines Agency (EMA) concluded that pioglitazone was useful in the treatment of type 2 diabetes mellitus as a second-line agent when metformin was not effective or contraindicated and that its use should be restricted in duration (<2 years), cumulative dose (<28,000 mg), and patients with bladder cancer risk [34]. Multiple studies followed the initial safety warning and have shown mixed results for the relationship between the increased risk of bladder cancer and the use of pioglitazone [35–47].

Hyperglycemia and hyperinsulinemia in people with diabetes are risk factors for cancer development because they can both induce aberrant cell proliferation [48, 49]. Insulin use has been linked to cancer risk although this finding is controversial [50, 51]. However, the molecular mechanisms and conditions that lead to carcinogenesis in urinary bladder epithelium coexpressing PPAR*α* and PPAR*γ* after exposure to metformin and pioglitazone in the presence of high glucose and high insulin are not known.

In this study, we investigated tumorigenesis under hyperglycemic and hyperinsulinemic conditions with pioglitazone plus metformin treatment using human bladder epithelial cell (HBlEpC). Furthermore, we examined the effects on cell viability, proliferation, and cell cycling as well as the associated signaling pathways to identify potential therapeutic targets.

## 2. Materials and Methods

### 2.1. Cell Culture and Reagents

Human bladder epithelial cell (HBlEpC) was purchased from Cell Applications (San Diego, CA, USA) and cultured in bladder epithelial cell growth medium (Cell Applications). Pioglitazone HCl, metformin HCl, and human recombinant insulin were purchased from Sigma-Aldrich (St. Louis, MO, USA).

### 2.2. Cell Viability and Proliferation Assay

Cell viability was evaluated using the 3-(4,5-dimethylthiazol-2-yl)-2,5-diphenyltetrazolium bromide (MTT) assay at 24, 48, and 72 h. HBlEpC was seeded in 96-well plates (5 × 10^3^ cells/well) and cultured in bladder epithelial cell growth medium containing 5.5 mM glucose (control; i.e., low-glucose condition), 55 mM glucose (high-glucose condition), or 55 mM glucose plus 0.5 mL (50 U) insulin (high glucose with insulin condition) with or without various doses of pioglitazone (2.5–25 *μ*M) or metformin (0.2–5 mM). The proliferation rate of HBlEpC was determined at 72 h using a cell proliferation enzyme-linked immunosorbent assay (ELISA) BrdU kit (Roche Diagnostics, Mannheim, Germany) according to the manufacturer's instructions.

### 2.3. Western Blot Analysis

Cells (2.5 × 10^5^ cells/mL) were cultured in six-well plates and cotreated with metformin, pioglitazone, and various concentrations of glucose and insulin. Cells were harvested and lysed in Nonidet P-40 buffer (Elpis Biotech, Daejeon, Korea) containing a protease inhibitor cocktail (Sigma-Aldrich). The total amount of protein in cell lysates was determined using the Micro BCA™ protein assay kit (Pierce, Rockford, IL, USA) and separated using 10% or 12% sodium dodecyl sulfate polyacrylamide gel electrophoresis (SDS-PAGE) and then transferred to a nitrocellulose membrane (Millipore, Billerica, MA, USA), which was blocked with 5% skim milk in Tris-buffered saline containing 0.1% Tween 20 for 1 h at room temperature (23.6 ± 3.8°C) and hybridized with primary antibodies (Cell Signaling Technology, Danvers, MA, USA) overnight at 4°C. Antibodies against the following proteins were used: *β*-actin, cyclin D, cyclin E, Cdk2, Cdk4, p53, p21, p15/16, c-Jun N-terminal kinase (JNK), phosphorylated- (phospho-) JNK (Thr183/Tyr185), p38 mitogen-activated protein kinase (MAPK), phospho-p38-MAPK (Thr180/Tyr182), extracellular signal-regulated kinase 1/2 (ERK1/2), phospho-ERK1/2 (Thr202/Tyr204, all from Cell Signaling Technology, Beverly, MA, USA), c-Myc, caveolin-1 (cav-1), RB, and phospho-RB (Santa Cruz Biotechnology, Santa Cruz, CA, USA). Bound antibodies were detected using horseradish peroxidase-conjugated secondary antibodies for 1 h at room temperature (23.6 ± 3.8°C), and immunoreactivity was detected using an enhanced chemiluminescence assay kit (Advansta, Menlo Park, CA, USA) according to the manufacturer's instructions. Protein bands were visualized using an LAS3000 luminescent image analyzer (Fujifilm Life Science, Tokyo, Japan).

### 2.4. Real-Time Polymerase Chain Reaction (PCR)

Total RNA was extracted using the TRIzol reagent (Thermo Fisher Scientific, Waltham, MA USA) according to the manufacturer's instructions and transcribed into cDNA using oligo (dT) primers and reverse transcriptase. Target cDNA was amplified using the following forward and reverse primer sequences: B cell lymphoma 2 (Bcl-2), 5′-GGG-TAT-GAA-GGA-CCT-GTA-TTG-G-3′ and 5′-CAT-GCT-GAT-GTC-TCT-GGA-ATC-T-3′ and Bcl-2-associated X protein (Bax), 5′-GGA-GCT-GCA-GAG-GAT-GAT-TG-3′ and 5′-AGT-TGA-AGT-TGC-CGT-CAG-A. The mRNA levels were quantitated using an ECO real-time reverse transcription- (RT-) polymerase chain reaction (PCR) system (Illumina, San Diego, CA, USA) and SYBR Green Master Mix kit (Takara, Tokyo, Japan) with the formula 2^−ΔΔCq^, where ΔΔCq is the difference between the threshold cycle of the target cDNA and an endogenous reference.

### 2.5. Statistical Analysis

Statistical analysis was performed using the GraphPad Prism v.5.01 software (GraphPad Inc., La Jolla, CA, USA). Data are expressed as the mean ± standard error of the mean. Differences between means were evaluated using the unpaired *t*-test, and a *p* < 0.05 was considered statistically significant.

## 3. Results

### 3.1. High Glucose with Insulin Enhances Viability and Proliferation in Cells Treated with Diabetes Drugs

**Figure 1:**
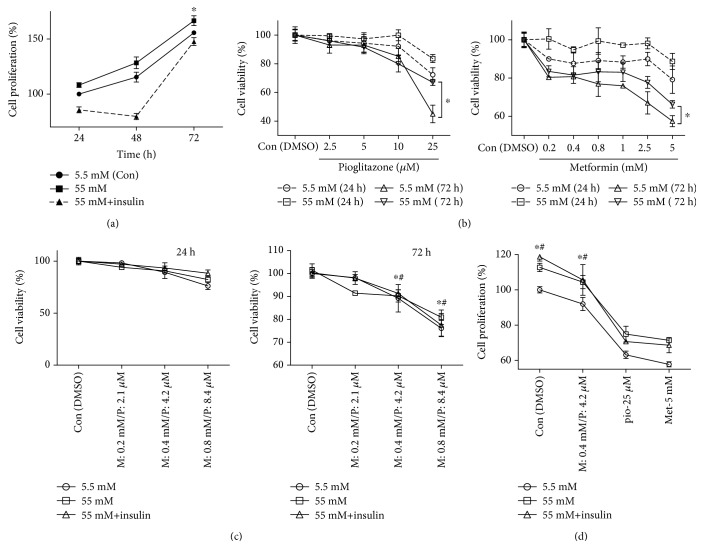
The effect of high glucose and insulin concentration on cell proliferation or viability of HBlEpC exposed to diabetes drugs using MTT assay. The cells were seeded in 96-well plates (5 × 10^3^ cells/well) and cultured with bladder epithelial cell growth medium containing low glucose or high glucose or high glucose with insulin. (a) The proliferation rate HBlEpC was assessed using MTT assay at low glucose (5.5 mM) or high glucose (55 mM) or high glucose with insulin (50 units). ^∗^*p* < 0.05, high glucose (55 mM) versus low glucose (5.5 mM). (b) Cytotoxic effect of pioglitazone and metformin on HBlEpC. The cells exposed to metformin (0.2, 0.4, 0.8, 1.0, 2.5, and 5 mM) or pioglitazone (2.5, 5, 10, and 25 *μ*M) under low- or high-glucose condition for 24 h or 72 h. Cell viability was determined by MTT assay. ^∗^*p* < 0.05, high glucose (55 mM) versus low glucose (5.5 mM) at 72 h. (c) The effect of glucose and insulin on HBlEpC viability cotreated with metformin (M) and pioglitazone (P) were analyzed by MTT assay. (d) Proliferation rates of HBlEpC exposed to metformin (M or Met) and pioglitazone (P or pio) were determined using BrdU incorporation at 72 h. ^∗#^*p* < 0.05, high glucose (55 mM) versus low glucose (5.5 mM). Data represent mean ± SD of three independent experiments. Results are representative of three independent experiments.

We performed BrdU incorporation and MTT assays to determine whether metformin and pioglitazone affect cell proliferation and viability under conditions of high glucose and insulin. The growth rate of HBlEpC was delayed in the presence of high glucose with insulin at 24 and 48 h; there was no difference in growth rate between cells treated with low and high glucose concentrations (5.5 and 55 mM, respectively) or high glucose with insulin at 72 h (Figure 1(a)). Metformin and pioglitazone reduced the cell viability in a dose- and time-dependent manner; however, the cytotoxic effects were attenuated under high-glucose conditions at 72 h (Figure 1(b)). Although the cytotoxicity against HBlEpC was slightly potentiated by combining metformin with pioglitazone in a dose- and time-dependent manner (Figure 1(c)), the proliferation of cells cotreated with metformin and pioglitazone and cultured in the presence of high glucose with insulin was significantly higher than that of cells treated with high doses of metformin or pioglitazone (Figure 1(d)). These results suggest that high glucose and insulin concentrations stimulate signaling pathways that promote survival and growth in bladder epithelial cells exposed to antidiabetic drugs.

### 3.2. High Levels of Glucose and Insulin Promote Cell Cycle Progression in Pioglitazone-, Metformin-, and Their Combination-Treated Cells

**Figure 2:**
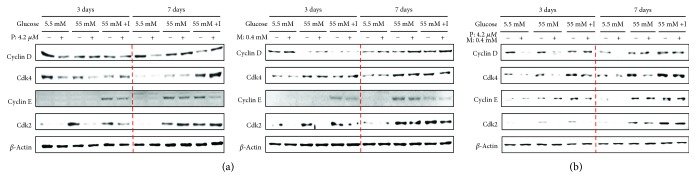
The activity of cyclin-Cdk complexes, which promote cell cycle progression in HBlEpC-treated diabetic drugs under high glucose or high glucose with insulin. The cells (2.5 × 10^5^ cells/mL) treated with metformin (M) or pioglitazone (P) and cotreated with both drugs were cultured with bladder epithelial cell medium containing low glucose or high glucose or high glucose with insulin in six-well plates. At days 3 and 7, the cells were harvested and then immunoblotting for cyclin D, cyclin E, and Cdk2/4 was carried out. Results are representative of three independent experiments.

We next investigated whether treatment with pioglitazone or metformin or combination of both drugs modulates cell cycle progression under conditions of high glucose and insulin. The activity of Cdk2 in cells treated with pioglitazone or metformin was suppressed under high-glucose condition and high glucose with insulin and that of Cdk4 also inhibited by pioglitazone in the presence of low or high glucose on day 3. Meanwhile, the treatment with pioglitazone or metformin has no effect on the level of cyclin D, Cdk4, cyclin E, and Cdk2 under high glucose and high glucose with insulin on day 7 (Figure 2(a)). Cdk2/Cdk4 activity and cyclin D expression were suppressed by cotreatment with pioglitazone and metformin on day 3 under low or high glucose. However, the expression of cyclin D and Cdk2/Cdk4 was recovered on day 7 under culture conditions containing high glucose or high glucose with insulin (Figure 2(b)). These results suggest that insulin combination under high-glucose conditions overrides cell cycle arrest induced by diabetes drugs in long-term cultures.

### 3.3. Prolonged Exposure to High Glucose and Insulin Concentrations Suppress Cdk Inhibitors in Diabetes Drug-Treated Cells

**Figure 3:**
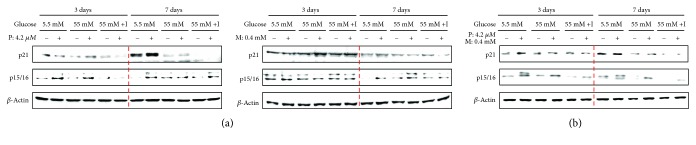
The effect of high glucose with insulin on cell cycle regulator protein p21 and p15/16 in diabetes drug-treated HBlEpC. The cells (2.5 × 10^5^ cells/mL) treated with metformin (M) or pioglitazone (P) and cotreated with both drugs were cultured with bladder epithelial cell growth medium containing low glucose or high glucose or high glucose with insulin in six-well plates for 3 days or 7 days. After treatment, the cells were harvested at indicated time and then immunoblotting for p21 and p15/16 were carried out. Results are representative of three independent experiments.

To determine whether high concentrations of glucose and insulin affect the levels of Cdk inhibitors, we examined the expression of the Cdk inhibitors p21 and p15/16 in drug-treated HBlEpC cultured in the presence of high glucose and insulin. The activity of p21 and p15/p16 was upregulated under low- or high-glucose conditions on day 3 after treatment with pioglitazone, but not the cell exposed to metformin (Figure 3(a)). However, the levels of p21 and p15/16 in drug-treated HBlEpC were suppressed after long-term culture in the presence of high concentrations of glucose and insulin (on day 7, Figure 3(a)). Although the p21 and p15/16 activation in HBlEpC was slightly enhanced on day 3 after cotreatment with pioglitazone and metformin under low- or high-glucose concentrations (on day 3, Figure 3(b)), prolonged exposure to high glucose with insulin significantly blocked the activity of p21 and p15/16 in HBlEpC cotreated with pioglitazone and metformin (on day 7, Figure 3(b)). These results suggest that glucose and insulin concentrations are more critical for cell cycle progression than drug treatment that negatively regulates Cdk inhibitors.

### 3.4. High Concentrations of Glucose with Insulin Induce c-Myc and Bax Expression in Pioglitazone- and Metformin-Treated Cells

**Figure 4:**
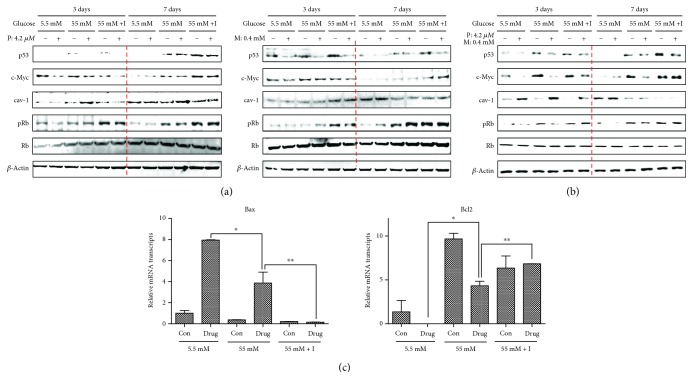
The expression of oncogenesis-related proteins and the activity of antiapoptotic Bcl-2 or proapoptotic Bax in drug-exposed HBlEpC. The cells (2.5 × 10^5^ cells/mL) treated with metformin (M) or pioglitazone (P) and cotreated with both drugs were cultured with bladder epithelial cell medium containing low glucose or high glucose or high glucose with insulin in six-well plates. (a) The cells were harvested at day 3 and day 7, and the expression of oncoproteins, including p53, c-Myc, caveolin-1 (cav-1), and RB was examined using western blot analysis. Results are representative of three independent experiments. (b) The cells (2.5 × 10^5^ cells/mL) cotreated with metformin and pioglitazone were cultured at same condition for 48 h. The levels of Bax or Bcl-2 mRNA transcript were determined by qPCR. Data represent mean ± SD of three independent experiments. Results are representative of three independent experiments. ^∗^*p* < 0.05, high glucose (55 mM) versus low glucose (5.5 mM). ^∗∗^*p* < 0.01, high glucose (55 mM) versus high glucose (55 mM) with insulin.

We also investigated the levels of tumor suppressors and protooncogenic proteins HBlEpC after treatment with pioglitazone or metformin to determine whether carcinogenesis occurs under high-glucose and insulin conditions. In single drug-treated HBlEpC, the expression of the tumor suppressor p53 was decreased on day 3 after exposure to high glucose and high glucose with insulin, whereas phosphorylated RB was increased under the condition of high glucose with insulin (Figure 4(a)). In addition, the expression of c-Myc protein and RB phosphorylation upregulated on day 7 in single drug-treated cells under high glucose with insulin (Figure 4(a)). The level of cav-1 was upregulated on day 3 in pioglitazone-exposed HBlEpC; this along with p53 expression was suppressed under a high-glucose environment (Figure 4(a)). However, long-term treatment with pioglitazone or metformin failed to enhance the tumor suppressor, *cav-1*, at high concentrations of glucose and insulin (Figure 4(a)). Furthermore, combined treatment with pioglitazone and metformin not only downregulated p53 expression but also upregulated the level of c-Myc, cav-1, and phosphorylated RB on day 3 in the presence of high glucose with insulin (Figure 4(b)). In addition, enhanced c-Myc and phosphorylated RB expression also detected at day 7 in cells cotreated with pioglitazone and metformin under high glucose with insulin (Figure 4(b)).

We next examined the mRNA levels of the apoptosis-related genes *Bax* and *Bcl-2* using quantitative real-time PCR in HBlEpC cotreated with pioglitazone and metformin under conditions of high glucose with or without insulin. The mRNA expression of the proapoptotic factor Bax was suppressed in drug-treated HBlEpC after 48 h exposure to high glucose with insulin compared to conditions of low or high glucose alone (Figure 4(c)). Furthermore, the transcript level of the antiapoptotic factor Bcl-2 was upregulated in drug-treated HBlEpC in the presence of high glucose with insulin (Figure 4(c)). These results suggest that high-glucose concentrations with insulin are critical for the activation of cell cycle-related oncoproteins and antiapoptotic genes for cell viability in diabetes drug-treated cells.

### 3.5. High Glucose and Insulin Blocked the Upregulation of Phospho-ERK and p38MAPK in Drug-Treated Cells

**Figure 5:**
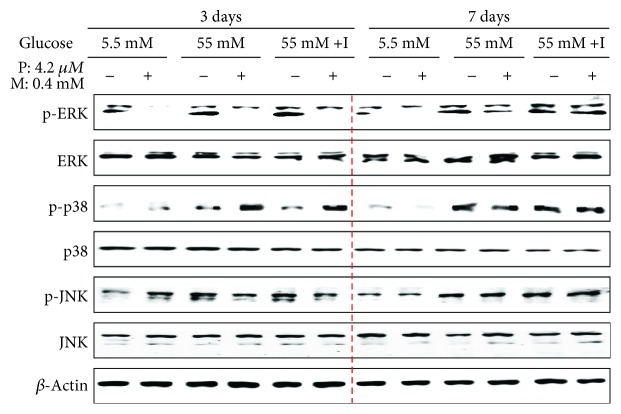
The activity of MAPK in diabetes drug-treated HBlEpC under high glucose with insulin condition. The cells (2.5 × 10^5^ cells/mL) cotreated with metformin (M) and pioglitazone (P) were cultured with RPMI-1640 containing low glucose or high glucose or high glucose with insulin in six-well plates for 3 days or 7 days. The cells were harvested, and then western blots for MAPK were performed. Results are representative of three independent experiments.

MAPKs respond to various stressors and regulate PPAR*γ* expression [52, 53]. The activation of the p38MAPK pathway induces p21, subsequently suppressing the levels of cyclin D-Cdk4 and cyclin E-Cdk2 complexes [54]. Based on these observations, we investigated whether cotreatment with pioglitazone and metformin of HBlEpC modulates MAPK activation at high concentrations of glucose and insulin. ERK phosphorylation was suppressed in drug-treated HBlEpC on day 3, whereas the level of phospho-p38MAPK in drug-treated cells was enhanced in the presence of high glucose with or without insulin (Figure 5, left panel). Although the level of phospho-ERK and p38MAPK was induced by high concentrations of glucose with or without insulin on day 7, cotreatment with pioglitazone and metformin failed to upregulate the expression of phospho-ERK and p38MAPK in HBlEpC compared to the levels of untreated cells (Figure 5, right panel). These results suggest that long-term exposure to high glucose and insulin inhibits the processes of p38MAPK-mediated cell cycle arrest in drug-treated bladder epithelial cells.

## 4. Discussion

Metformin is a biguanide commonly administered to patients with type 2 diabetes mellitus to reduce blood glucose levels and protect against cancer by inducing cancer cell apoptosis [55, 56]. TZDs are synthetic PPAR*γ* ligands that modulate metabolic homeostasis and improve glucose tolerance in diabetes by increasing insulin sensitivity [57]. However, long-term activation of PPAR*γ* can lead to weight gain, fluid retention, and osteoporosis [58]. In addition, results from clinical trials of these agents suggest an increased risk of cardiovascular-related diseases including congestive heart failure and myocardial infarction (MI) [59]. Although pioglitazone does not have the same cardiovascular risks as rosiglitazone, it may increase the risk of bladder cancer [29–31] by unknown mechanisms. A study by Yang et al. showed that pioglitazone decreased the expression of p53 in urothelial transitional epithelium cells and bladder cancer cells under long-term culture condition. However, the effect of pioglitazone in diabetic condition is still not defined [60]. We demonstrate here that high-glucose and insulin concentrations not only increased cell viability and proliferation but also triggered aberrant cell cycle progression and expression of protooncogenic proteins in HBlEpC exposed to pioglitazone and metformin. These results suggest that uncontrolled high serum levels of glucose and insulin are a more critical factor in the cell cycle regulation and oncogenic protein expression of bladder epithelium than long-term use of pioglitazone and metformin is.

The p21 and phosphorylated RB protein levels regulate the proliferation and differentiation of myoblasts [61]. Activation of G1 phase cyclin-Cdk complexes results in RB phosphorylation [62]. Expression of the Cdk inhibitors p21 and p15/16 is associated with cell cycle arrest by the suppression of cyclin-Cdk activity and RB phosphorylation, whereas Cdk-mediated phosphorylation of RB also abrogates the function of p21 [63]. High glucose and insulin supplementation—for example, with insulin-like growth factor 1—increases the expression of cyclins A, B1, and D1 and p21-Cdk4 complexes [64]. TZDs do not affect the transcription of cyclins or Cdks but prevent mitogen-induced degradation of p21, thereby inhibiting cyclin D-Cdk4 and cyclin E-Cdk2 activation [65]. Furthermore, the stimulation of PPAR*γ* by activated RB leads to cell cycle arrest by the regulation of p21 and cyclin D1 [24–26]. These results suggest that stimulation with synthetic PPAR*γ* ligands accompanied by high glucose and insulin levels can have conflicting effects on cell cycle progression and carcinogenesis. The elevated Cdk4 and Cdk2 levels induced by high-glucose concentrations in HBlEpC was downregulated on day 3 after cotreatment with pioglitazone and metformin. Furthermore, cyclin D-Cdk4 and cyclin E-Cdk2 activation was increased in HBlEpC on day 7 even after cotreatment with pioglitazone and metformin in the presence of high glucose and insulin. These results suggest that prolonged exposure to high glucose and insulin reversed cell cycle arrest in HBlEpC stimulated with TZDs.

DNA-damaging agents activate p21 expression induced by p53, leading to G1 arrest and inhibition of Cdk4 and Cdk2 activities [66–68]. c-Myc overexpression promotes tumorigenesis by inducing DNA double-strand breaks and preventing escape from the cell cycle [69, 70] and along with increased c-Myc gene copy number is frequently observed in bladder cancer [71]. Although there is no clear association between alterations in p53 and c-Myc protein expression [71], p53 is a positive transcriptional and translational regulator of cav-1, whose expression is downregulated in p53-inactivated fibroblasts [72]. The levels of c-Myc and cav-1 in HBlEpC exposed to pioglitazone and metformin were expressed in an opposing manner on day 3 under hyperglycemic conditions despite decreasing the p53 expression. Prolonged exposure to high glucose with or without insulin for 7 days also suppressed p53 and cav-1 expression, whereas the levels of c-Myc were upregulated in HBlEpC exposed to pioglitazone and metformin. These results suggest that oncogenic signaling pathways could be reactivated by the induction of c-Myc and inhibition of the tumor suppressor functions of p53 and cav-1 in diabetes drug-treated HBlEpC cells under long-term hyperglycemic and hyperinsulinemic conditions.

MAPK signaling mediates the growth factor function of insulin in hyperglycemia [73]. In humans and animals with high glucose levels, muscle tissues normally respond to insulin stimulation by ERK activation [74]. In contrast, synthetic TZDs regulate inflammatory and immune responses by inhibiting MAPK signaling [75]. Phospho-p38MAPK regulates the cell cycle by suppressing cyclin E-Cdk2 activation [76]. Cotreatment with bortezomib and celecoxib was found to induce p38MAPK-dependent cyclin D inhibition and cell arrest in p53-deficient cancer cells [77]. Although the levels of activated p38MAPK and p21 were upregulated in drug-treated HBlEpC in the presence of high glucose, phospho-p38MAPK expression was not prominently increased on day 7 in drug-treated HBlEpC compared to the levels of untreated cells under conditions of high glucose with or without insulin. These results suggest that phospho-p38MAPK in drug-treated HBlEpC fail to inhibit cyclin E-Cdk2 and cyclin D-Cdk4 activation under hyperglycemic/hyperinsulinemic conditions and long-term exposure to high glucose with insulin overrides the inhibitory effects of activated MAPK-mediated cell cycle arrest in diabetes drug-treated HBlEpC.

Glucose absorption by glucose transporter (GLUT) plays an important role in cell proliferation. Although GLUT1 and GLUT3 is closely associated with bladder cancer progression, there are no specific reports about the role or relationship between the GLUT and carcinogenesis of bladder [78, 79]. Enhanced reactive oxygen species (ROS) production under high glucose inhibits the proliferation of human pancreatic *β* cells by elevation of p21, inhibitor of cyclin D-Cdk4 [80]. To date, it has not been studied how hyperglycemic conditions affect GLUT expression and intracellular ROS production during the development of bladder cancer. Based on these previous studies, we will further investigate whether high-glucose concentration has influences on GLUT expression and ROS production in carcinogenesis of bladder epithelium.

Chronic hyperglycemia and hyperinsulinemia are known to increase cancer risk [81, 82]. Furthermore, our data provide insight into how these conditions enhance the carcinogenic effect in drug-treated bladder epithelial cells and indicate that uncontrolled hyperglycemia and hyperinsulinemia in people with diabetes are probably more dangerous than TZD administration or PPAR*γ* stimulation in a clinical situation.

## Data Availability

The data used to support the findings of this study are included within the article.
